# HLA-DRB1 May Be Antagonistically Regulated by the Coordinately Evolved Promoter and 3′-UTR under Stabilizing Selection

**DOI:** 10.1371/journal.pone.0025794

**Published:** 2011-10-18

**Authors:** Benrong Liu, Yonggui Fu, Zhifen Wang, Sisi Zhou, Yu Sun, Yuping Wu, Anlong Xu

**Affiliations:** State Key Laboratory of Biocontrol, Guangdong Province Key Laboratory of Pharmaceutic Functional Genes, Department of Biochemistry, College of Life Sciences, Sun Yat-sen (Zhongshan) University, Guangzhou, People's Republic of China; The University of Hong Kong, China

## Abstract

HLA-DRB1 is the most polymorphic MHC (major histocompatibility complex) class II gene in human, and plays a crucial role in the development and function of the immune system. Extensive polymorphisms exist in the promoter and 3′-UTR of HLA-DRB1, especially a LTR (Long terminal repeat) element in the promoter, which may be involved in the expression regulation. However, it remains unknown how the polymorphisms in the whole promoter region and 3′-UTR to regulate the gene expression. In this study, we investigated the extensive polymorphisms in the HLA-DRB1 promoter and 3′-UTR, and how these polymorphisms affect the gene expression in both independent and jointly manners. It was observed that most of the haplotypes in the DRB1 promoter and 3′-UTR were clustered into 4 conserved lineages (H1, H2, H3 and H4), and showed high linkage disequilibrium. Compared with H1 and H2 lineage, a LTR element in the promoter of H3 and H4 lineage significantly suppressed the promoter activity, whereas the activity of the linked 3′-UTR increased, leading to no apparent difference in the final expression product between H1/H2 and H3/H4 lineage. Nevertheless, compared with the plasmid with a promoter and 3′-UTR from the same lineage, the recombinant plasmid with a promoter from H2 and a 3′-UTR from H3 showed about double fold increased luciferase activity, Conversely, the recombinant plasmid with a promoter from H3 and a 3′-UTR from H2 resulted in about 2-fold decreased luciferase activity. These results indicate that the promoter and 3′-UTR of HLA-DRB1 may antagonistically regulate the gene expression, which may be subjected to stabilizing selection. These findings may provide a novel insight into the mechanisms of the diseases associated with HLA-DRB1 genes.

## Introduction

Major histocompatibility complex (MHC) plays a crucial role in the regulation of immune response. Since the landmark discovery of MHC restricted T-cell recognition by Zinkernagel and Doherty [1], Numerous studies have concentrated on how T-cells are trained to recognize self-peptide∶self-MHC complex (pMHC) by positive and negative selection in thymus [2]–[4]. Both the avidity of the T-cell receptor (TCR) to the pMHC and the density of pMHC on the thymic cortical epithelial cell contributes to the consequence of the positive and negative selection for T-cells [5]. Although previous studies have revealed that the concentration of self-peptide ligands and their affinity to TCR may influence the outcome of positive selection and negative selection [6]–[7], but very limited studies were reported on how the amount of pMHC affects the consequences of positive or negative selection for thymocytes. Patients lacking MHC class II, known as the bare lymphocyte syndrome (BLS), were subjected to severe immune deficiency, and they were devoid of peripheral CD4+ T-cells [8]. Additionally, it was shown that decreased expression of HLA-DR molecules in thymoma may affect on CD4+ T-cell development [9]. Analysis based on BLS patients suggested that abnormal MHC II expression level may impair CD4+ T-cell development. Apart from this, MHC-II molecules also play very important roles in the native immune response. Deficiency of MHC-II attenuates the Toll-like receptors (TLRs) triggered inflammation [10].

HLA-DR molecules are constitutively expressed on the cell surface of B lymphocytes, thymic cortical epithelial cells, dendritic cells (DCs), monocytes and other antigen presenting cells (APC), with the function of presenting antigens T-cells [11]–[12]. DR molecules function as the heterodimer consisted of DR alpha (encoded by DRA locus) and DR beta chains (encoded by DRB loci, including *DRB1*, *DRB3*, *DRB4* and *DRB5*), HLA-DRA is monomorphic, whereas HLA-DRB, especially DRB1, is highly polymorphic [13]–[14]. Among the MHC II members, HLA-DR plays the most crucial role in the human immune system since it accounts for more than 90% of the HLA class II isotypes expressed on antigen-presenting cells [14]. Because the HLA-DRB1 genes located on different DR haplotypes are vastly diversified, and some DR haplotypes also comprise another DRB encoding gene except for DRB1, so 2–4 very distinct HLA-DR heterodimers can be formed in an individual depending on the DR haplotypes [15]. The expression level of DRB alleles determines the amount of a variety of functional DR heterodimers on the surface of APCs, which may in turn influence the quantity and ratio of mature T-cells with different specificity. In other words, the expression regulation of HLA-DR molecules is very important for CD4+ T-cells selection and activation during the adaptive immune response. Since the expression level of HLA-DRB1 loci was demonstrated to be much higher than that of other HLA-DRB coding loci (*DRB3*, *DRB4* and *DRB5*) [16]–[17], and HLA-DRB1 was also the most reported MHC gene in association with different diseases [18]–[26]. The regulation of HLA-DRB1 expression, the dominant locus which expresses HLA-DR, must be under delicate control. Previous studies revealed that the polymorphisms in the core promoter of HLA-DRB1 gene caused a significant change of the promoter activity [17], [27]–[31]. However, the mechanism underlying this effect is still unknown. Polymorphisms in regulatory cis-elements may impact on the phenotypic outcomes in a highly variable and unpredictable fashion [32]. Many retro-elements, such as LINEs (long interspersed nuclear elements), SINEs (short interspersed nuclear elements) and LTRs (long terminal repeats), could play important roles in the regulation of gene expression [33]. In the past decades, 3′-UTR was also demonstrated to contain important cis-elements for gene expression regulation. It is very popular that distinct length of 3′-UTRs are produced by alternative polyadenylation (APA) in different cell types and cell stages, regulating the final product of gene expression [34]. To understand the evolution of the regulatory region of HLA-DRB1 and assess the functional consequence of the polymorphisms in these regions, we sequenced about 4.6 kb of HLA-DRB1 promoter region including 5′-UTR and 2.6 kb of 3′ non-coding region (3′-NCR) containing entire 3′-UTR from 26 normal Han Chinese, and used the dual luciferase reporter gene assay system to experimentally determine the effect of the polymorphisms in HLA-DRB1 promoter and 3′-UTR on gene expression. This study first explored how the polymorphisms in the promoter and 3′-UTR of HLA-DRB1 to regulate the gene expression.

## Results

### Most of the Haplotypes in HLA-DRB1 Promoter and 3′-UTR were Grouped into Four Distinct Lineages

**Figure 1:**
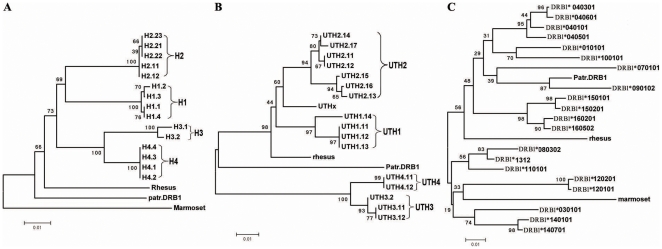
Phylogenetic Tree Constructed with Various Parts of HLA-DRB1 Sequence. The taxon names that designated with H plus numbers represent haplotypes from the promoter of DRB1 gene, and UTH plus numbers represent haplotypes from 3′-UTR of DRB1 gene. The 1^st^ number denotes the lineage which comprises all the sequences clustered on the same clade, and the numbers after the dot represent sublineages and haplotypes, respectively. UTHx signifies the haplotype that is distinct from the others. *Rhesus*, part.DRB1 and *marmoset* indicates that the corresponding sequence come from DRB1 gene of *rhesus*, *chimpanzee* and *marmoset*, respectively. All bootstrap values are shown on the nodes. A: Phylogenetic tree was constructed with the 5′-proximal promoter sequences. B: 3′-UTR tree. C: Tree was constructed with exon2 sequences.

**Figure 2:**
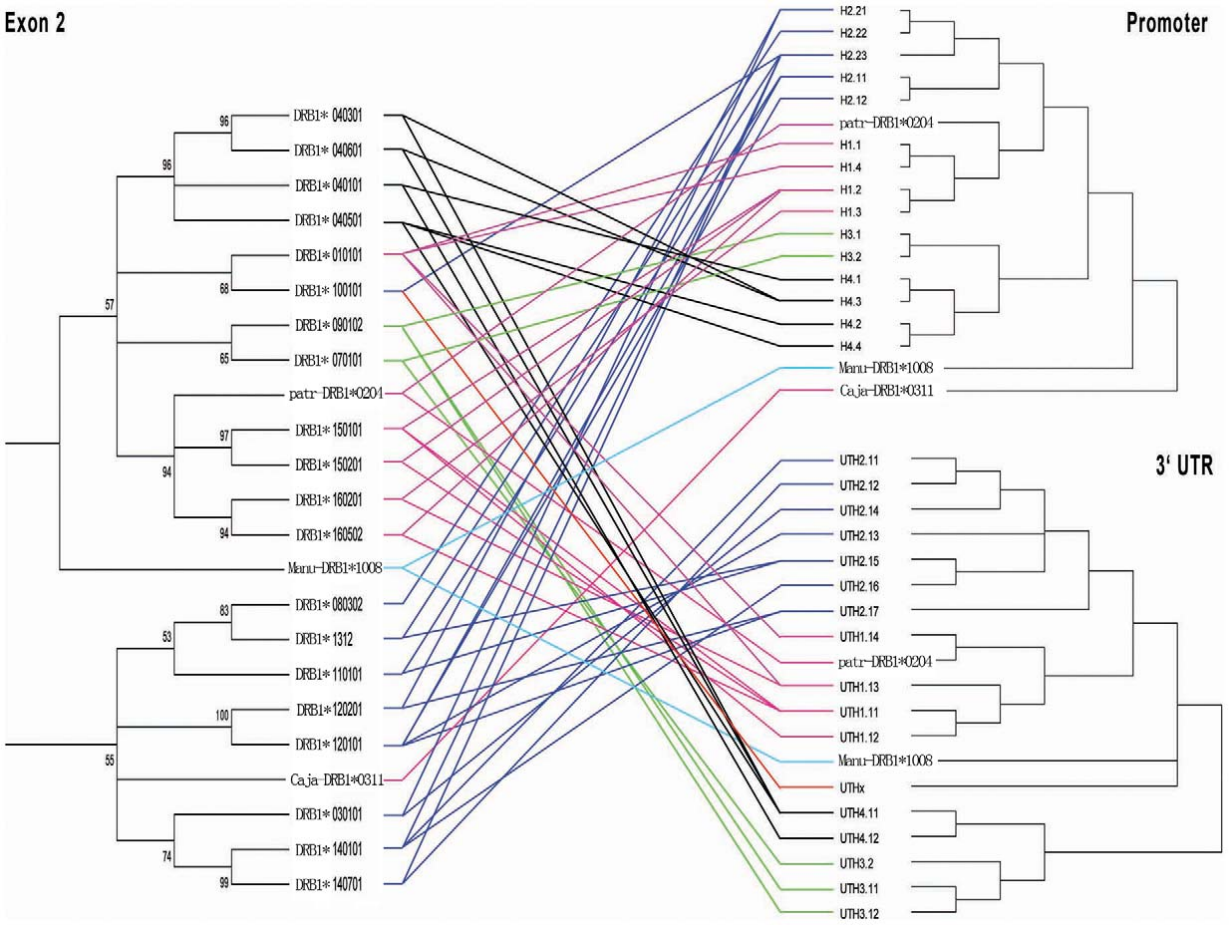
Linkage Relationship between Promoters, Exon2 and 3′-UTRs. Branch lengths for all trees are arbitrary, and Bootstrap values larger than 50% are shown on the nodes. The three parts located on the same haplotype were connected with lines. The different color lines denote the deeply separated lineages.

A total of 51 promoter sequences and 50 3′-UTR sequences were obtained, and the new sequences were submitted to GenBank (http://www.nlm.nih.ncbi.org) and assigned a GenBank accession number of *FJ464430* to *FJ464461*. Phylogenetic analysis showed that the sequences from both promoter and 3′-UTR were clustered apparently into 4 deep clades, which were designated as H1, H2, H3 and H4 for promoter clades (Figure 1A), and UTH1, UTH2, UTH3 and UTH4 for 3′-UTR (Figure 1B), respectively. One unique 3′-UTR haplotype denoted as UTHx was derived from HLA-DRB1*10:01:01 (Figure 1B). Linkage analysis between promoters, exon2 and 3′-UTRs revealed four tightly linked lineages, namely H1-UTH1-exon2 (DR01, DR15 and DR16), H2-UTH2-exon2 (DR03, DR08, DR11, DR12, DR13 and DR14), H3-UTH3-exon2 (DR07 and DR09) and H4-UTH4-exon2 (DR04) (Figure 2). The evolutionary relationship among all haplotypes except for UTHx is very similar on the 3′-UTR tree and the 3′-UTR downstream (3′-UTR-DS) tree. The UTHx was clustered with UTH2 lineage on the 3′-UTR tree, but it is more ancient on the 3′-UTR-DS tree (Figure S1).

**Figure 3:**
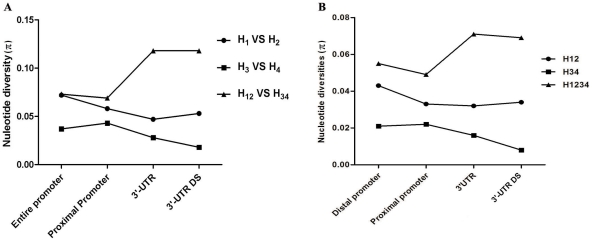
Comparison of Nucleotide Diversity between Different Lineages or lineage combinations in Various Regions. H12 represents a lineage combination with lineage 1 and lineage 2; H34 denotes a lineage combination with lineage 3 and lineage 4; H1234 indicates a lineage combination with all lineages. A: The nucleotide diversity was calculated by averaging the polymorphic site within the compared lineages. B: The average nucleotide diversity (per site per sequence) was computed.

There are extreme differences among lineages but low diversity within lineages in both promoter and 3′-UTR. The frequency spectrum analysis showed that the most moderate frequency alleles exist in the DRB1 promoters (Figure S2) and the similar distribution of a frequency spectrum is observed for 3′-UTR (data not shown). When we performed Tajima's D test and Fu and Li's test by taking account for all lineages together or only one lineage, a notable difference was observed (Table S1). When we considered all lineages together, the nucleotide diversity (π) are 0.05053 and 0.07263 in the promoter region and 3′-UTR, respectively. In promoter region, Tajima's D is 2.43186 (*P*<0.05 ). Fu and Li's D* test statistic is 2.09095 ( *P*<0.02) and Fu and Li's F* test statistic is 2.73971 (*P*<0.02). In 3′-UTR, Tajima's D is 2.3799 with a statistical significance *P*<0.05, Fu and Li's D* and F* is 1.8129 and 2.4305, respectively, and both with a statistical significance (*P*<0.02; Table S1). The above results suggested that the promoter and 3′-UTR may have been subjected to balancing selection. However, when we only selected the sequences grouping in certain lineage or sub-lineage for analysis, low nucleotide diversity was observed. For H3 lineage, the nucleotide diversity (π) is 0.00083. Tajima's D is −2.37453 (*P*<0.001 ). Fu and Li's D* test statistic is −3.26961 ( *P*<0.02) and Fu and Li's F* test statistic is −3.47765 (*P*<0.02). Tajima's D, Fu and Li's D* and F* test for UTH1 are −1.84699, −2.50331 and −2.65713, respectively (*P*<0.05) (Table S1). These results indicated that the polymorphism patterns within H3 and UTH1 lineage may have been shaped by purifying or positive selection. The nucleotide diversity between H12 (H1 and H2) and H34 (H3 and H4) in the 3′-UTR region is significantly higher than that in the promoter region. However, the nucleotide diversity between H1 and H2, or H3 and H4, in the 3′-UTR region is slightly lower than that in the promoter region (Figure 3). The above indicated that the 3′-UTR diverged faster between H12 and H34 than the promoter, suggesting the 3′-UTR was subjected to strong positive selection after the separation of the DRB1 lineage of H12 and H34.

At least one SNP was observed in each of the cis-elements such as TATA box, CAAT box, Y box, X box and W box (Figure S3). We compared the SNP density in these cis-elements, their flanking sequences from −1 to −200 bp, as well as 5′-UTR among 4 DRB1 promoter lineages. SNP density is 72.5 SNPs/kb in the regulatory boxes, 110.0 SNPs/ kb in flanking sequences and 45.0 SNPs/ kb in 5′-UTR, respectively. The SNP density in flanking sequences is higher than that in the regulatory boxes and 5′-UTR, suggesting the regulatory boxes and 5′-UTR were subjected to purifying selection.

### The Promoter Activity of HLA-DRB1 Decreased in the Absence of Its Linked 5′-UTR

**Figure 4:**
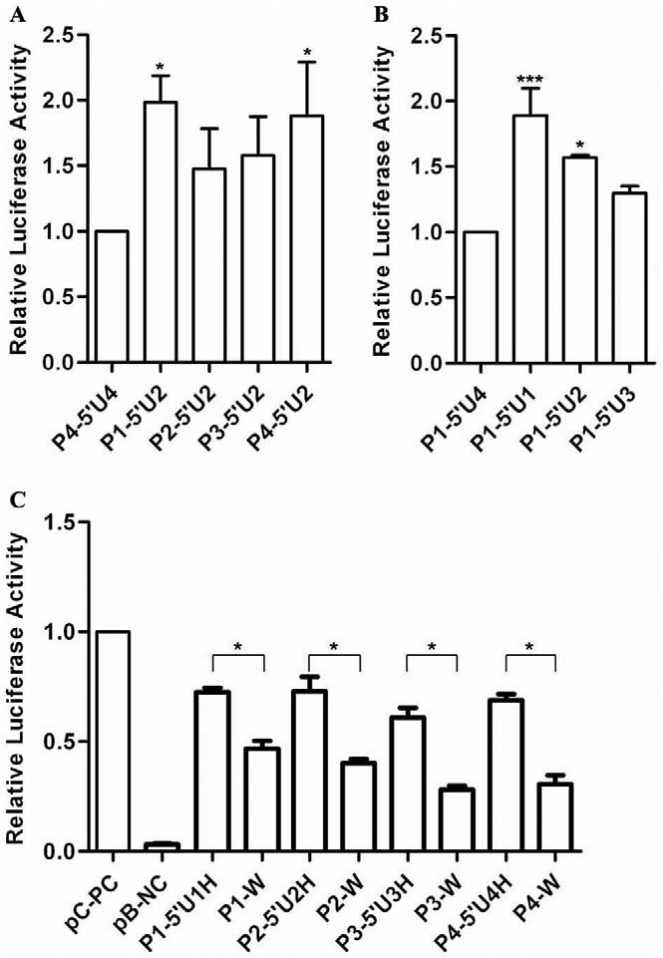
Relative Luciferase Activity of Constructs with HLA-DRB1 Derived Promoter and 5′-UTR Sequences. A: luciferase activity was tested for the plasmids into which the different DRB1 lineage derived promoter sequences with lineage 2 derived 5′-UTR were inserted. B: Luciferase activity was tested for the plasmids into which the promoter sequences from DRB1 lineage 1 with different lineage-derived 5′-UTR were inserted. C: Luciferase activity was tested for the plasmids into which the different DRB1 lineage-derived promoter sequence with or without its 5′-UTR were inserted. Firefly luciferase activity of each sample was normalized by *Renilla* luciferase activities. The P4-5′U4 (A), P1-5′U4 (B) and pGL3.0 control vectors (C) were used as controls, and the normalized luciferase activities of controls were set as relative activity 1. Therefore no error bars were shown for the P4-5′U4, P1-5′U4 and pGL3.0 control vector. The relative luciferase activity of the other samples was calculated by their normalized luciferase activity to that of the normalized controls. Error bars represent standard error of the mean estimated from three separate experiments. *, *P*<0.05; ***, *P*<0.001, compared with the control or comparison between two groups as indicated.

Dual luciferase reporter system was used to analyze the expression activity of different promoter constructs. Whereas no significant variation in luciferase expression level was observed when the reporter gene was placed under the control of different promoters together with the same 5′-UTR from H2 lineage (Figure 4A). We found a remarkable effect of different 5′-UTRs on the luciferase level. The expression of luciferase (P1-5′U1, P1-5′U2, P1-5′U3 and P1-5′U4) driven by the same promoter sequence derived from H1 lineage and different 5′-UTRs obtained from H1, H2, H3 and H4 lineages dropped increasingly, and statistic significant differences were found between P1-5′U1 and P1-5′U4, and between P1-5′U2 and P1-5′U4 (*P*<0.05), suggesting the polymorphisms in the 5′-UTR could affect the promoter activity (Figure 4B). We also investigated the influence of the DRB1 promoter with or without 5′-UTR on the expression levels. Analysis for the four paired corresponding promoter constructs showed that the DRB1 promoter with its linked 5′-UTR had much higher transcription activity than that without 5′-UTR in Raji cells (Figure 4C). However, no significant difference was observed among the constructs (P1-5′U1H, P2-5′U2H, P3-5′U3H and P4-5′U4H) that include constitutive promoter and 5′-UTR or among the constructs (P1-W, P2-W, P3-W and P4-W) that include only DRB1 promoter sequence without 5′-UTR sequence (Figure 4C). These results indicate that the polymorphisms in the proximal promoter (−1 to −535 bp) of HLA-DRB1 gene by themselves do not impact on the promoter activity. However, the polymorphisms in the 5′-UTR may play an important role through coordinately regulating the gene expression with those in the promoter region.

### A LTR Element Located at the Distal Promoter of H3 and H4 Lineage Led to the Repression of Promoter Activity

**Figure 5:**
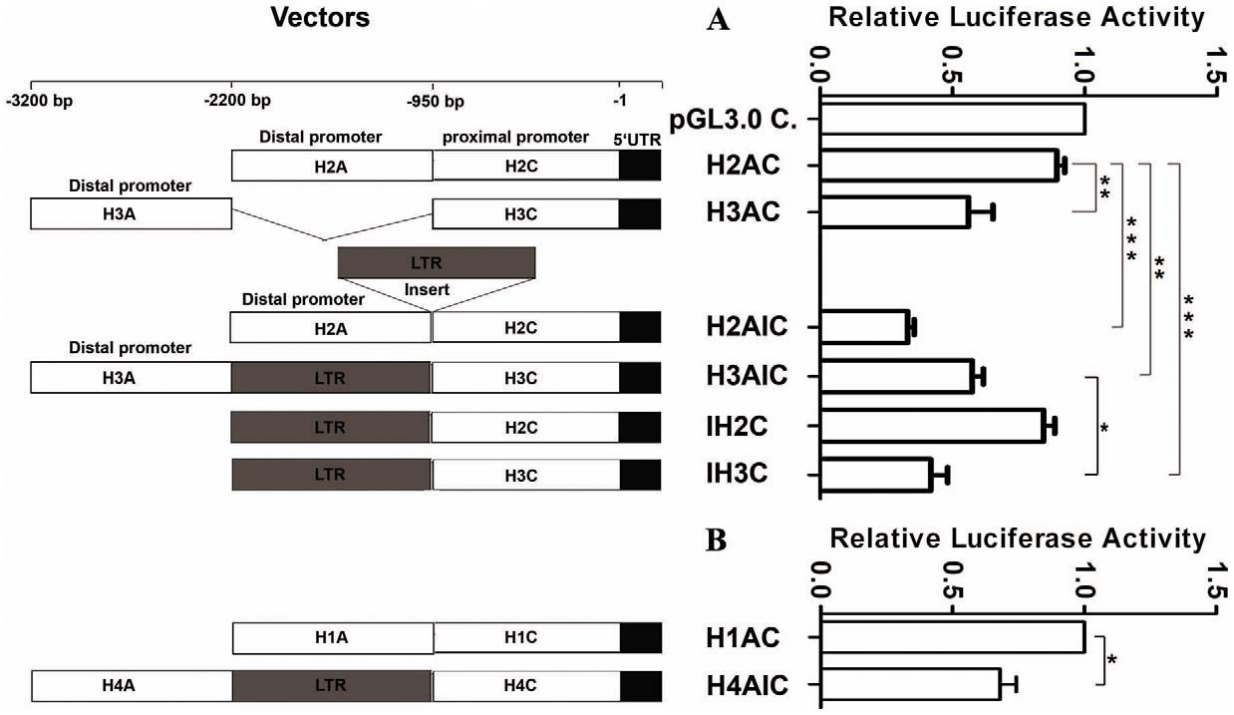
Relative Luciferase Activity under the Control of DRB1 Promoters with or without a LTR Insert. The strategy for constructing the promoter vectors is shown on the left and the relative luciferase activity of corresponding vectors is shown on the right. H1, H2, H4 and H4 represent the sequences derived from promoter lineage H1, H2, H3 and H4 of DRB1, and A, C denotes the distal promoter segment and proximal promoter segment, respectively. “I” denotes the insert LTR actually located at H3 lineage about 950 bp upstream from the transcription start site. Firefly luciferase activity of each sample was normalized by *Renilla* luciferase activity. The pGL3.0 control vector (A) and H1AC (B) is used as controls, and the normalized luciferase activity of control was set as relative activity 1. Therefore no error bar was shown for pGL3.0 control vector and H1AC. The relative luciferase activity of the other samples was calculated by their normalized luciferase activity to that of the normalized control. Error bars represent standard error of the mean estimated from three replicate experiments. *, *P*<0.05, **, *P*<0.01; ***, *P*<0.001, comparison between two groups as indicated.

The insertion of repetitive elements into the gene promoter region usually affects the promoter activity. In order to explore the potential influence of the LTR element in the H3 and H4 lineages on activity of their promoters, we created constructs by cloning the long promoter region (from +66 bp to −3200 bp) from each DRB1 lineage into pGL3.0 basic vector, and simultaneously constructed several recombinants by inserting the LTR into the promoter sequence from H2 lineage. Compared with the constructs of H3AIC (from H3 lineage) and H4AIC (from H4 lineage) which comprise a LTR element, the long promoter constructs H1AC (from H1 lineage) and H2AC (from H2 lineage) which contain no LTR element showed significantly increased expression activity (*P*<0.01) (Figure 5). Furthermore, the expression activity of a construct (H2AIC) by inserting a LTR element derived from H3 lineage greatly decreased (*P*<0.001) (Figure 5). The above results suggest the insertion of LTR element significantly impact on the promoter activity. However, deletion of the LTR element in a construct H3AC could not apparently increase the promoter activity, and yet the transcription activity of a construct (IH3C) that excluding the upstream part of the LTR was significantly decreased (*P*<0.05) (Figure 5). Another construct IH2C was recombined with the proximal promoter from H2 lineage and a LTR element, unexpectedly, whose transcriptional activity was not decreased compared with that of H2AC (Figure 5). These findings indicate that the influence of the LTR on promoter activity was depended on the sequence environment, and may act through altering the secondary or tertiary structures required for the binding of promoter complex.

### Polymorphisms in the 3′-UTR of HLA-DRB1 Counteract the Influence of that in Its Linked Promoter on Expression

**Figure 6:**
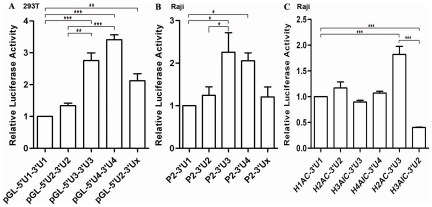
Luciferase Activity was Detected for the 3′-UTR from Different HLA-DRB1 Lineage. A: Luciferase activity was detected in 293T cells for the plasmids into which different DRB1 lineage-derived 3′-UTRs were inserted downstream the reporter gene. B: Luciferase activity was detected in Raji cells for the plasmids into which a same DRB1 promoter sequence and different DRB1 lineage derived 3′-UTRs were simultaneously cloned up and downstream of the reporter gene, respectively. C: Luciferase activity was detected in Raji cells for the plasmids into which the naturally linked long promoter sequence and 3′-UTR from DRB1 lineages were simultaneously cloned up and downstream of the reporter gene, respectively. The construct of H2AC-3′U3 represented the plamids that the promoter and 3′-UTR of the reporter gene were derived from DRB1 lineage 2 and lineage 3, respectively. But the construct of H3AIC-3′U2 was just reverse to H2AC-3′U3. Firefly luciferase activity of each sample was normalized by *Renilla* luciferase activity. The pGL-5′U1-3″U1 (A), P2-3′U1 (B) and H1AC-3′U1 (C) is used as controls, and the normalized luciferase activity of control was set as relative activity 1. Therefore no error bar was shown for pGL-5′U1-3″U1, P2-3′U1 and H1AC-3′U1. The relative luciferase activity of the other samples was calculated by their normalized luciferase activity to that of the normalized control. Error bars represent standard error of the mean estimated from three replicate experiments. * *P*<0.05, ** *P*<0.01 and *** *P*<0.001 are indicated as the comparative significancce between two groups.

In order to find out whether the polymorphisms in 3′-UTR of HLA-DRB1 are involved in the regulation of gene expression, the luciferase activities with various 3′-UTR constructs were determined in 293T and Raji cells. A significant higher luciferase activity was observed for the 3′-UTR from DRB1 lineages of UTH3 and UTH4 than that from DRB1 lineages of UTH1, UTH2 and UTHx (Figure 6A). Similar results were obtained when different DRB1 3′-UTR sequences from various lineages were placed in the constructs containing the same HLA-DRB1 promoter and 5′-UTR sequence originated from lineage H2, (Figure 6B). The above results showed that the effect of the polymorphisms in 3′-UTR on the expression regulation is reverse to that in the promoter from same lineages. That being the case, then similar luciferase activity should be observed among the constructs in which the luciferase gene was placed under the control of both 3′-UTR and LP from the same lineage. This point was experimentally demonstrated in Raji cells (Figure 6C). Moreover, apparently increased luciferase activity was observed with the recombinant plasmid (H2AC-3′U3) in which the luciferase gene was placed under the control of 3′-UTR from H3 lineage and LP from H2 lineage, and opposite changes was obtained for the recombinant plasmid H3AIC-3′U2 in which the luciferase gene was placed under the control of 3′-UTR from H2 lineage and LP from H3 lineage (Figure 6C). These results indicate that the changes in expression of the HLA-DRB1 gene caused by the polymorphisms in promoters can be counteracted by that in their linked 3′-UTRs, it is strongly suggested that HLA-DRB1 gene have been subjected to stabilizing selection in the evolutionary history.

## Discussion

In previous studies, it was reported that polymorphisms in cis-elements located in the core promoter of HLA-DRB1 genes led to apparent expression difference [17], [27]–[31]. However, in this study, no significant difference in promoter activity was observed between 4 distinct promoter lineages within a region of about 600 bp, though in which polymorphisms almost reside in every cis-element. Moreover, the promoter activity was influenced by the polymorphism in 5′-UTR only as they linked with the same promoter sequence (Figure 4B). The above phenomenon suggested that the sequence out of the core promoter (from TIS to about −300 bp) also contributes much to the promoter activity, and the promoter activity of different HLA-DRB1 lineages may be affected by the antagonistic action among polymorphisms in the whole promoter region. Inhibition of the promoter activity by the LTR element in upstream of −950 indicated that it is hard to suppose the promoter activity for different DRB1 lineage by investigating its core promoter region.

It is very interesting that the impact of the polymorphisms in the 3′-UTR on the gene expression is just reverse to that in the promoter, suggesting antagonistic regulation between the promoter and 3′-UTR of HLA-DRB1 gene. This regulatory mechanism ensures the 4 distinct HLA-DRB1 lineages, although with different promoter activity, may have a similar final expression product. So we proposed that it may be very important to maintain an identical expression level for two distinct HLA-DRB1 alleles in an individual, and HLA-DRB1 gene was subjected to stabilizing selection in the evolutionary history.

Stabilizing selection between the alleles from distinct DRB1 lineages suggested that the maintenance of equal expression of two distinct DRB1 alleles in an individual must have a very important biological significance. Although the expression of most genes in primate and model organisms was demonstrated to be subjected to stabilizing selection [35]–[38], yet it was poorly understood for the evolutionary mechanism. In this study, we experimentally demonstrated a typical mechanism for stabilizing selection, which was dependent on the antagonistic coevolution between the promoter and 3′-UTR. However, what's the biological significance for maintaining the relatively stable expression among the distinct DRB1 lineages? In previous studies, it was revealed that the ratio variation of MHC II I-A^g7^ gene product to I-A^k^ product in mixed MHC *mice* determined the difference in autoproliferative response, and the higher ratio would cause more autoreactive T-cells to evade negative selection into the periphery and readily elicit autoimmune response [39]. Inefficient presentation of Self-peptide to T-cells in the thymus causes a part of T-cells evading from the negative selection and entering into the periphery, which is one of the hypothetic mechanisms for autoimmune disease demonstrated experimentally [39]–[40]. Lessons from the bare lymphocyte syndrome (BLS) told us that the quantity of MHC II molecules play key roles in thymocytes selection [41]. HLA-DRB1 is the most extensively studied MHC II genes, which are constitutively expressed in B lymphocytes, dendritic cells and macrophage cells, and is induced to express in other cells [42]. Many previous studies concluded that the polymorphisms in the cis-elements of HLA-DRB1 promoters could greatly affect the promoter activity [17], [27]–[28], which led to a puzzling question of what would have happened if the various promoters lead to differential expression of two distinct HLA-DRB1 alleles in an individual. Not only does the positive selection need interaction with the MHC-peptide complex (pMHC), but also does the negative selection [7]. Therefore, the types of MHC and its quantities on the cell surface could affect the selection results for mature T-cells. Maintaining the appropriate ratio of gene products of two heterozygous alleles may be critical for T-cells selection and activation, which could interpret why the polymorphisms in the DRB1 3′-UTR is implicated in the expression regulation on the opposite effect of the polymorphisms in the DRB1 promoter. Therefore, balancing the mature T-cell types may be the fundamental biological significance for the stabilizing selection acting on the expression of HLA-DRB1 genes, which may provide new insight for the research on why DRB1 genes were associated with so many diseases.

The promoter regions of MHC II genes were subjected to Balancing selection [43]–[44]. In this study, extreme diversity among lineages observed in HLA-DRB1 regulatory region suggested a balance selection. However, very limited polymorphism within a lineage could not be explained by balance selection. Moreover, each promoter and 3′-UTR lineage are linked with many diversified exons, implicating that the regulatory region and the exons of HLA-DRB1 gene were subjected to different natural selection. The limited polymorphisms in the promoter regions within a lineage might partly be attributed to the reduction of diversity by recombination. Nevertheless, no recombination signal was observed between the lineages. It was well accepted that the exon 2 of HLA-DRB1 gene have been subjected to balancing selection in its evolutionary history [45]. The exon 2 of HLA-DRB1 gene encodes antigen recognition site (ARS) and peptide binding site (PBS), so high diversity in this region implies that it may present more extensive exogenous antigens, and the populations could be protected against more pathogens [46]–[47]. Moreover, it is beneficial for populations maintaining the diversity in exon 2 of DRB1 by balanced selection [48]–[49]. When plague was prevalent in certain periods, some special alleles which could present the antigen in the most efficient way would be selectively survived. And in this circumstance, positive selection played a major role. Because of a microorganism evolving very fast, the ARS and PBR were necessarily driven to evolve faster than any other part of the gene [50]. As it was proposed in previous works, the pathogens may be the main force which drives and maintains the remarkable polymorphism in the MHC [51]–[52]. Anyway, the polymorphic pattern in exon 2 of DRB1 gene is the consequence of ongoing balanced selection and infrequently positive selection. Since our results showed that the corresponding promoter lineage and 3′-UTR lineage were linked tightly, and they cooperatively worked to keep the similar expression among different DRB1 lineages, suggesting that most mutation occurred in the two regions would be deleterious. The fact that SNP density in cis-elements and 5′-UTR is lower than in their flanking regions also indicates that purifying selection have acted on DRB1 upstream regulatory region. Therefore, the complicated polymorphism pattern in the promoter and 3′-UTR of HLA-DRB1 must have been attributed to the hitch-hiking by the exons which are subjected to balancing selection, positive selection or other natural selection.

The nucleotide diversities between lineage 12 (lineage 1 and lineage 2) and lineage 34 (lineage 3 and lineage 4) in the 3′-UTR region is much higher than that in the promoter region, suggesting that the 3′-UTR and the promoter were subjected to different natural selection. The 3′-UTR from lineage 3 and 4 might be subjected to positive selection so as to evade the suppression to counteract the inhibition of its promoter activity. UTHx belongs to DRB1*10:01:01 which was recombined with the allele on the DR52 haplotype in intron 1 and has the most recent common ancestor with the alleles on DR51 and DR52 haplotype [53]. The position of UTHx haplotype on the 3′-UTR tree and 3′-UTR-DS tree suggested that the 3′-UTR have been subjected to stronger natural selection than the downstream sequence. Most part of the above may be contributed by the selective pressure of maintaining the stable expression of the different HLA-DRB1 gene.

In conclusion, the expression of HLA-DRB1 may be antagonistically regulated by its coordinately evolved promoter and 3′-UTR under stabilizing selection. The polymorphic pattern in the DRB1 promoter and 3′-UTR was shaped by multiple natural selection, such as purifying selection, positive selection, stabilizing selection, and hitch-hiking by the polymorphic sites in the exons. The findings in this study implicated that balancing the expression of two different MHC II alleles in an individual may be very important, which provide a novel insight into the pathogenic mechanism of MHC involving in many diseases.

## Materials and Methods

### Samples

Whole blood samples were collected from 26 Han Chinese volunteers from the different provinces. A written informed consent was obtained from each participant, and this study was conducted with the approval of the Institute Research Medical Ethics Committee of Sun Yat-Sen University. Genome DNA was extracted with Blood DNA kit (D3392-02) purchased from OMEGA Bio-tek, Inc., Guangzhou, China.

### Primer design

We downloaded 3 HLA-DRB1 reference sequences which located at different DR haplotypes, namely DR51 (DRB1*15:01:01, GenBank accession no. NG_002432), DR52 (DRB1*03:01:01, GenBank accession no. NG_002392), and DR53 (DRB1*04:01:01, GenBank accession no. NG_002433). Sequence alignment was performed with ClustalX 1.81, and all primers were designed with primer premier 5.0. The primer pairs and their relative positions were shown in Figure S4. Also, the sequencing primers were designed and most of which were lineage specific. All the primers were synthesized by Invitrogen (Shanghai, China) and the primer sequences were shown in Table S2.

### Long fragment amplification and investigation of linkage relationships

The amplification was performed in a 50 µl of reaction system containing 5 µl of 10×LA buffer, 0.25 mM of dNTP, 0.24 µM of each primer, 20 ng of genome DNA and 2.5 U of TakaRa LA Taq polymerase in which pfu enzyme was supplemented according to 1∶19. A total of 35 cycles run in the PCR after preheating at 92°C for 3 minutes, and for each cycle with denaturing at 95°C for 15 seconds, annealing at x °C (the “x” denotes the annealing temperature that varies according to different primer pairs) for 30 seconds and extension at 68°C for y (the “y” means the extension time for amplifying different length of fragments) minutes, then elongating at 68°C for 10 minutes. The PCR products were cleaned with Gel extraction Kit (OMEGA Bio-tek, Inc., Guangzhou, China), then sequenced directly and finally cloned into T-easy vector (Promega Corporation, Madison, WI).

To investigate linkage relationship among promoter, exon 2, and 3′-UTR, we designed 2 universal primer pairs (and) to amplify the fragment containing promoter region and exon 2, as well as the fragment containing 3′-UTR and exon 2 (Figure S4). Then the fragments were cloned into T-easy vector, and one positive clone was sequenced from two ends with sequencing primers T7 and SP6 for each segment. The end sequences were aligned with the known promoter sequences, exon 2 sequences and 3′-UTR sequences, respectively. Genotyping for exon 2 of HLA-DRB1 gene was according to the description of Kotsch [54]. We could get the linkage relationship between promoter regions and 3′-UTRs easily through analyzing the overlapping exon 2.

### Sequencing

The allele specific PCR product was purified by gel extraction with DNA Gel Extraction Kit (OMEGA bio-tek, Inc., Guangzhou, China) followed by sequencing with haplotype specific primers (Table S2) on an ABI 3730 sequencer using BigDye Terminator V3.0 cycle sequencing kit. The plasmid was extracted with Plasmid Mini kit (OMEGA bio-tek, Inc., Guangzhou, China) followed by sequencing. All different sequences were submitted to NCBI and the GenBank accession numbers were given as FJ464430 to FJ464461.

### Construction of expression vectors

The promoter lineages H1, H2, H3 and H4 were defined according to the clusters of the promoter sequences (Figure 1A). H1 represents the promoter sequences derived from DRB1*01, DRB1*15 and DRB1*16; H2 indicates the promoter sequences from DRB1*03, DRB1*08, DRB1*10, DRB1*11, DRB1*12, DRB1*13 and DRB1*14; H3 denotes the promoter sequences from DRB1*09 and DRB1*07, and H4 indicates the promoter sequences from DRB1*04. In order to compare the influence of different promoter lineages of HLA-DRB1 and their 5′-UTR on the gene expression level, we constructed 3 kinds of vectors by inserting DRB1 promoter derived fragments with or without 5′-UTR into pGL3.0 Basic vectors. The first kind of promoter constructs is that the luciferase gene was placed under the control of different promoters from distinct DRB1 lineages and fused with the 5′-UTR from lineages H2. We denominated these vectors as P1-5′U2, P2-5′U2, P3-5′U2 and P4-5′U2 (P1-4 represent the promoter derived from promoter lineages H1-4, and the “5′U2” indicates a 5′-UTR from promoter lineage H2). The fragment, including the promoter and 5′-UTR sequence, were amplified from lineage H4 was cloned into pGL3.0 basic vector as a control (P4-5′U4) (Figure S5A). The second kind of promoter constructs contains the same promoter sequence amplified from H1 lineage and various 5′-UTRs from distinct DRB1 lineage, which were denoted as P1-5′U1, P1-5′U2, P1-5′U3 and P1-5′U4, respectively. In these two constructs, the fusion fragment of promoter sequence and 5′-UTR were inserted into the *Hind* III and *Nco* I site, so they do not contain the 5′-UTR of luciferase reporter gene from the vector itself (Figure S5B). The third kind of promoter vectors was constructed by inserting the PCR segments from different DRB1 lineages containing with or without the 5′-UTR into the *Kpn* I and *Sma* I sites, which were designated as P1-5′U1H, P2-5′U2H, P3-5′U3H, P4-5′U4H, P1-W, P2-W, P3-W and P4-W, respectively. The former 4 vectors contain the 5′-UTRs, and the later 4 vectors are absent from the DRB1 derived 5′-UTR. (Figure S5C).

One LTR (MER11C) element is about 1.1 kb, which was inserted into the promoter region of H3 and H4 lineages upstream at the position −938 to −2065. Here, we defined the position of LTR elements as indel site, and use an “A” represent the distal promoter that located upstream of the indel site, an “I” indicates the LTR elements, a “C” denotes the proximal promoter that located between translation start site and indel site, and H1-4 represent the 4 distinct promoter lineage. To investigate whether the LTR element influences on the transcription activity of the promoter, we constructed 4 constitutive promoter vectors designated as H1AC, H2AC, H3AIC and H4AIC, respectively. Simultaneously, we constructed four recombinant plasmids namely H2AIC, IH2C, H3AC and IH3C, respectively (shown on the left of Figure 5).

We amplified the fragment containing the complete 3′-UTR and about 300 bp long downstream sequence from the poly(A) signal site from each 3′-NCR lineage of HLA-DRB1, which is about 586–594 bp long. We denoted the fragments of 3′-UTR from UTH1, UTH2, UTH3, UTH4 and UTHx as 3′U1, 3′U2, 3′U3, 3′U4 and 3′Ux, respectively, and constructed them into pGL3.0 Basic vector or pGL3.0 control vector between *Xba* I and *BamH* I site. We successfully constructed four different kinds of vectors and confirmed its fidelity by sequencing. 1) The vector in which the 3′-UTR and 5′-UTR of the luciferase gene were simultaneously substituted for that from different DRB1 lineages (Figure S6A). 2) The luciferase gene was placed under the control of the promoter from H2 lineage and the 3′-UTR from different DRB1 lineages (Figure S6B). 3) The luciferase gene was placed under the control of the long promoter and 3′-UTR from the same DRB1 lineage, and simultaneously created two recombinant plasmids in which the luciferase gene was placed under the control of the long promoter from H2 lineage (H2AC) and the 3′-UTR from UTH3 lineage (3′U3), as well as from H3 lineage (H3AIC) and the 3′-UTR from UTH2 lineage (3′U2).(Figure S6C).

### Transfection and luciferase assay

All plasmids for transfection were extracted with Qiagen Midi Kit-25 (Valencia CA, USA) according to manufacturer's instruction. The concentration of each construct was measured with Spectrophotometer ND-1000. Burkitt's lymphoma B cell line Raji was propagated with RPMI 1640 culture medium supplemented with 10% fetal bovine serum (FBS), 100 U/ml penicillin, and 100 µg/ml streptomycin. All promoter constructs and an internal control (pRL-TK vector) were co-transfected into Raji cells by electroporation, and into 293T cells with lipofactamine2000 (Invitrogen). The electroporation was performed as following: the mix of 200–400 µl cells and plasmids were transferred into a 4 mm cuvette pre-cooled on ice and discharged for 25 ms at 160 V on a Genepulser Xcell (Bio-Rad). Then, the cells were transferred to wells containing 2 ml RPMI 1640 complete culture medium and incubated at 37°C in a CO2 incubator with moist ambient air for 36–48 hours. The procedure of transfection with lipofactamine2000 was according to the manufacturer's instruction.

Transfected cells were harvested and schizolysed with passive lysis buffer attached with the Dual-Luciferase reporter assay system (Promega). Then, the enzyme activity of the reporter gene including the internal control was assayed with the Dual-Luciferase reporter assay system on a chemoluminescence meter (Monolight 3010C, Pharmingen). The fluorescence intensity representing promoter activity of each lineage was normalized by internal control and comparison was performed among all lineages.

### Data analysis

Sequence assembly was performed manually with BioEdit version 7.0 and the sequence alignment was carried out with clustalX 1.81. The polymorphism level, π (the average number of pairwise nucleotide differences per site) and θw (the number of segregating sites per nucleotide), was calculated with DnaSP version 4.50.3 [55]. Phylogenetic trees were constructed by MEGA3.1 [56] using the distance matrix calculated by Kimura's two-parameter method and the neighbor-joining method. The reliability of the NJ trees was assessed by bootstrapping with 1,000 pseudosamples.

Data for analysis of promoter activity were expressed as the mean ± standard error of mean (s.e.m.) from at least 3 separate experiments. Unless otherwise noted, the differences between groups were analyzed using Student t test when only 2 groups, or assessed by one-way ANOVA when more than 2 groups were compared. All tests performed were two-sided. Differences were considered statistically significant at *P*<0.05.

## Supporting Information

Figure S1 **Phylogenetic Analysis for the 3′-UTR and 3′-UTR Downstream Sequences (3′-UTR-DS).** Taxon names that designated with UTH plus numbers represent haplotypes containing 3′-UTR or 3′-UTR downstream sequences. UTHx signifies that the haplotype is distinct from the others. The arrow indicated the different position of the UTHx haplotype on the 3′-UTR tree and 3′-UTR-DS tree. The chimpanzee and rhesus sequences were used as reference sequences. The haplotypes belonging to the same lineage were denoted with opposite bracket.
(JPG)

Figure S2 **Slide Window Analysis for Nucleotide Polymorphisms and Frequency Spectrum Analysis for the Promoter Sequences of HLA-DRB1.** A mutation of size i means that is occurs i times in our sample of 52 sequences. The thin line represents the expected frequency spectrum in neutral equilibrium, and the black bars denote the observed values.
(JPG)

Figure S3 **Polymorphism in the Core Promoter Region of Partial DRB Alleles from** ***human*** **,** ***chimpanzee*** **,** ***rhesus*** **and** ***marmoset*** **.** The numbering is relative to the transcription start site. The cis-element box was encircled with a pane and the core sequence in a cis-element box was marked with underline.
(JPG)

Figure S4 **The Sketch of the Relative Position of Primers at HLA-DRB1 Gene.** The number – represents the primers at certain position, for example, represents the 5 primers designated as H1ASP1R, H21ASP1R, H22ASP1R, H3ASP1R and H4ASP1R which are specific for different sequence lineage. The distance marked in the figure denotes the approximate size of PCR product which varying for different lineage.
(JPG)

Figure S5 **The Sketch Map of Construction of HLA-DRB1 Promoter Vectors.** Only the reporter gene and its 5′ flanking region in which the multiple clonal sites are located, were shown on the sketch. The numbers in the parenthesis following the names of restriction endonuclease (REN) denote the relative positions of the RENs at the vector sequence. The rectangles below multiclonal sites represent the fragments of promoters or 5′UTRs from HLA-DRB1 genes, which have been inserted into the REN sites denoted by triangles. Three different kinds of promoter vectors were constructed and shown on A, B and C, respectively.
(JPG)

Figure S6 **The Sketch Map of Construction of HLA-DRB1 3′UTR and recombinant Vectors.** Only the reporter gene and its 5′ flanking region in which the multiple clonal sites are located were shown on the sketch. The numbers in the parenthesis following the names of restriction endonuclease (REN) denote the relative positions of the RENs at the vector sequence. The rectangles below multiclonal sites represent the fragments of promoters or 3′UTRs from HLA-DRB1 genes, which have been inserted into the REN sites denoted by triangles. Three different kinds of vectors were constructed and shown on A, B and C, respectively.
(JPG)

Table S1 **Neutral test in DRB1 promoter region and 3′ non-coding region with our data.**
(XLS)

Table S2 **All primer sequences involved in this paper.**
(XLS)
