# Supplementary figures and images for: HLA-DRB1 May Be Antagonistically Regulated by the Coordinately Evolved Promoter and 3′-UTR under Stabilizing Selection
Source: PLoS One. 2011 Oct 18;6(10):e25794. doi: 10.1371/journal.pone.0025794 (PMC3196528; doi:10.1371/journal.pone.0025794)

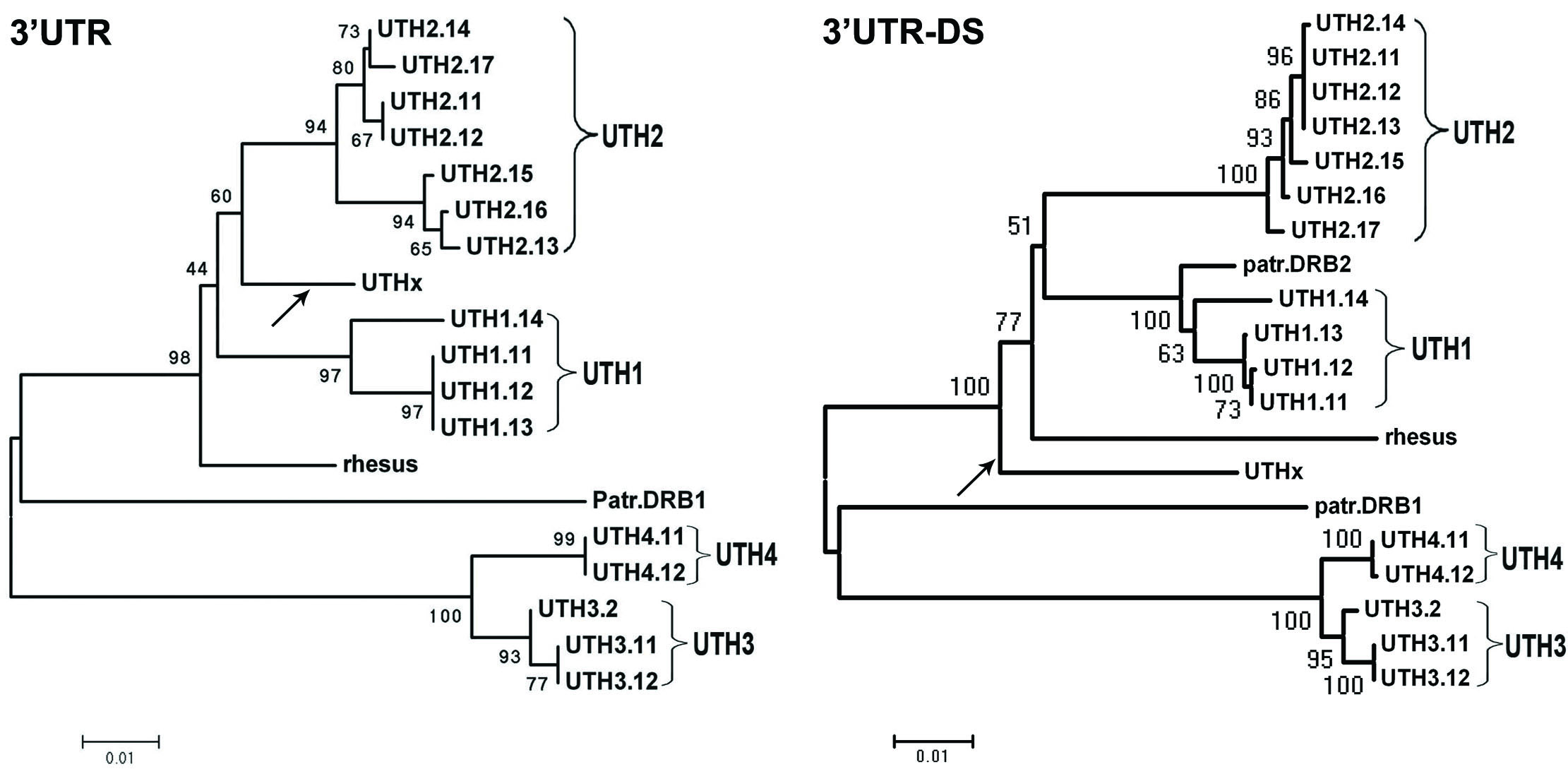

Supplement: Figure S1 — Phylogenetic Analysis for the 3′-UTR and 3′-UTR Downstream Sequences (3′-UTR-DS). Taxon names that designated with UTH plus numbers represent haplotypes containing 3′-UTR or 3′-UTR downstream sequences. UTHx signifies that the haplotype is distinct from the others. The arrow indicated the different position of the UTHx haplotype on the 3′-UTR tree and 3′-UTR-DS tree. The chimpanzee and rhesus sequences were used as reference sequences. The haplotypes belonging to the same lineage were denoted with opposite bracket. (JPG) [file pone.0025794.s001.jpg]

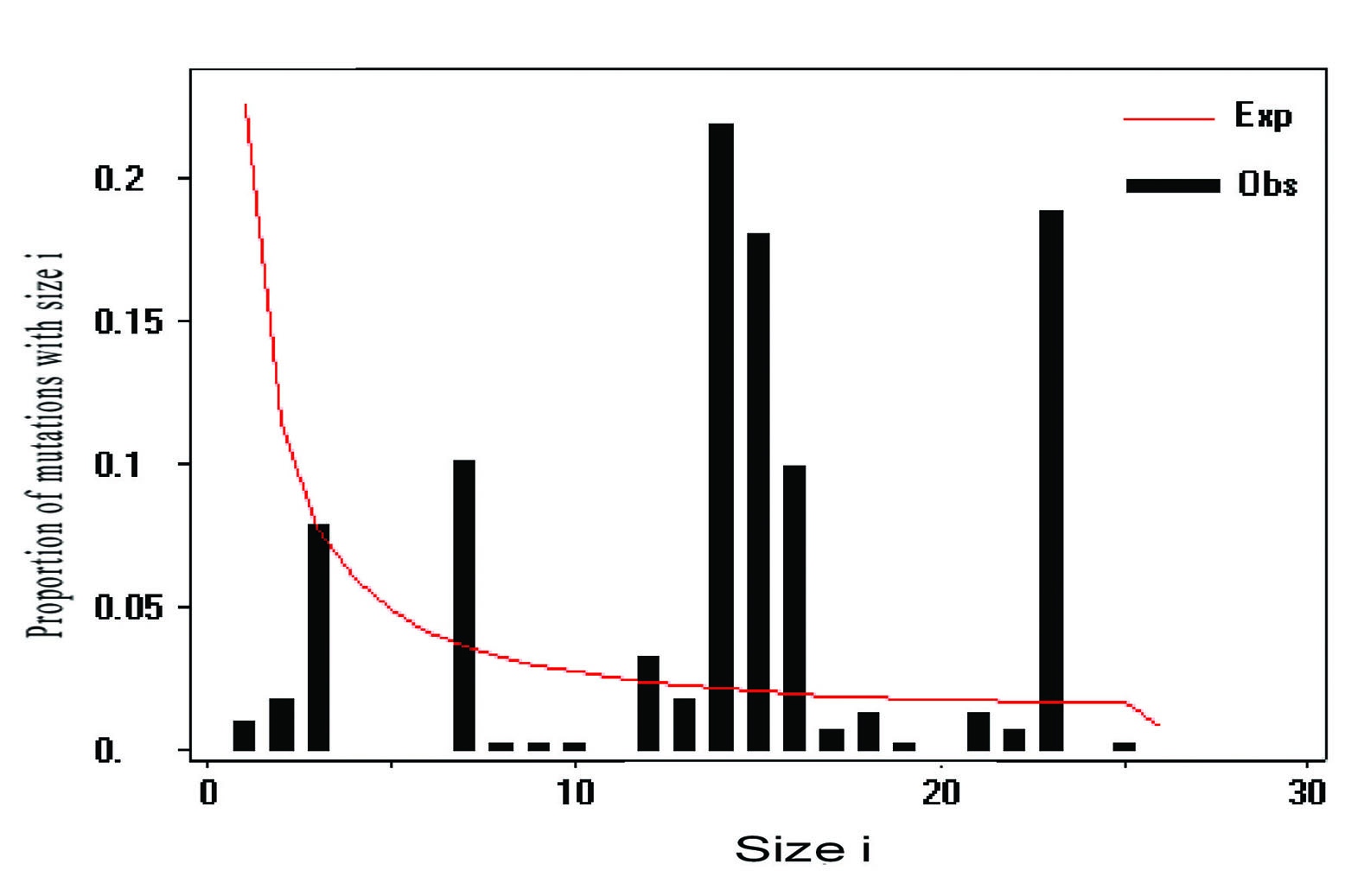

Supplement: Figure S2 — Slide Window Analysis for Nucleotide Polymorphisms and Frequency Spectrum Analysis for the Promoter Sequences of HLA-DRB1. A mutation of size i means that is occurs i times in our sample of 52 sequences. The thin line represents the expected frequency spectrum in neutral equilibrium, and the black bars denote the observed values. (JPG) [file pone.0025794.s002.jpg]

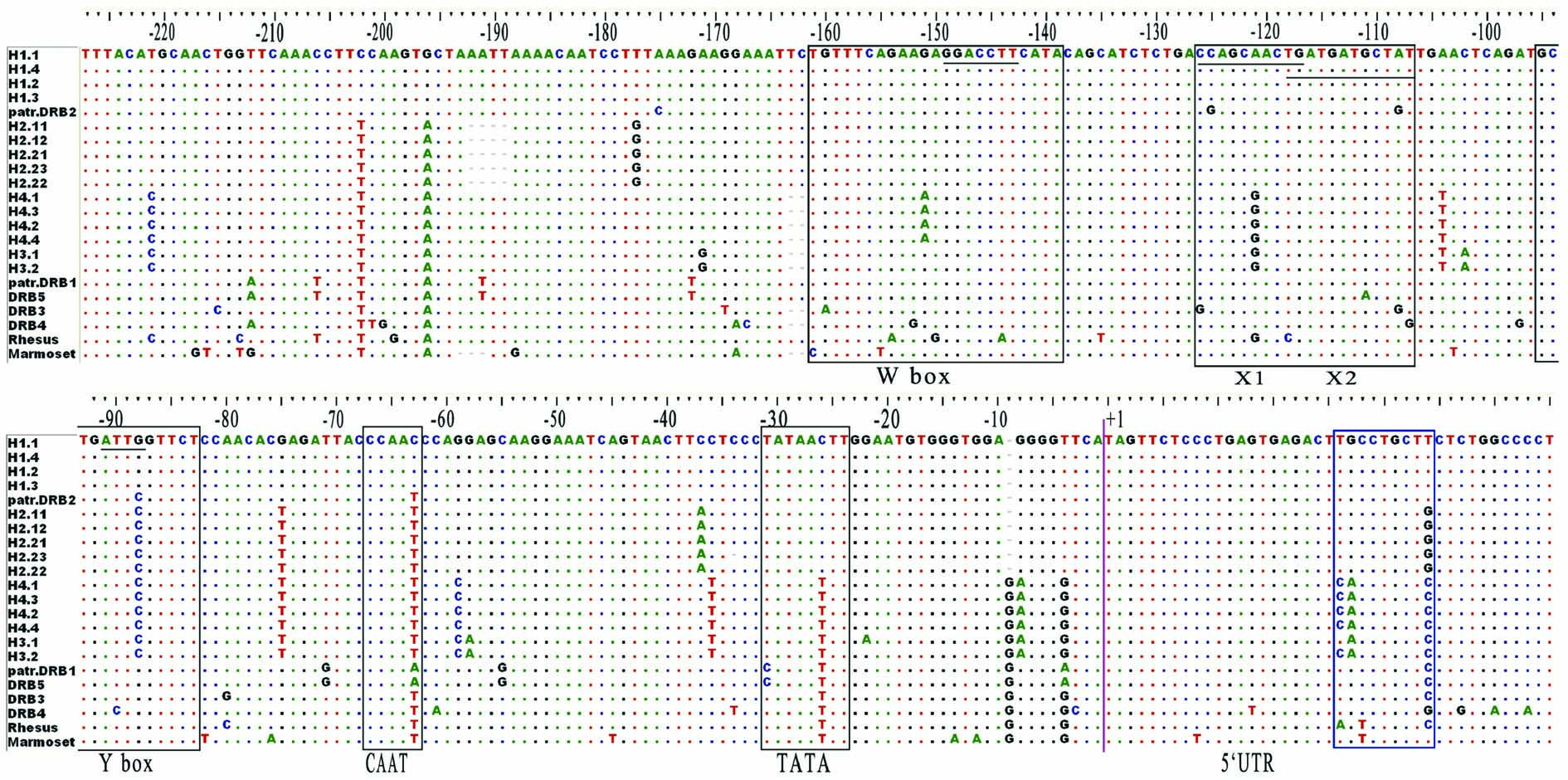

Supplement: Figure S3 — Polymorphism in the Core Promoter Region of Partial DRB Alleles from human , chimpanzee , rhesus and marmoset . The numbering is relative to the transcription start site. The cis-element box was encircled with a pane and the core sequence in a cis-element box was marked with underline. (JPG) [file pone.0025794.s003.jpg]

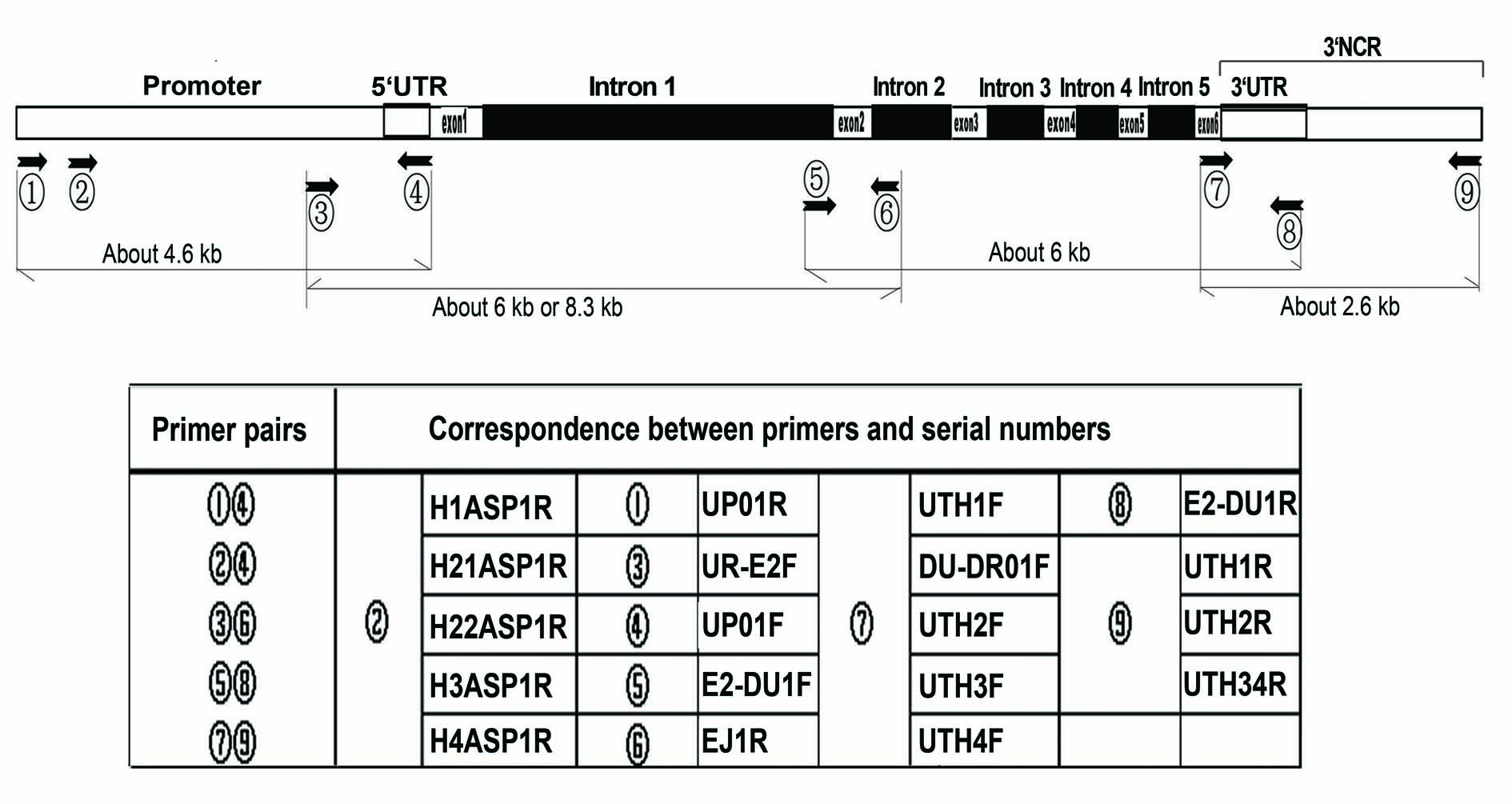

Supplement: Figure S4 — The Sketch of the Relative Position of Primers at HLA-DRB1 Gene. The number – represents the primers at certain position, for example, represents the 5 primers designated as H1ASP1R, H21ASP1R, H22ASP1R, H3ASP1R and H4ASP1R which are specific for different sequence lineage. The distance marked in the figure denotes the approximate size of PCR product which varying for different lineage. (JPG) [file pone.0025794.s004.jpg]

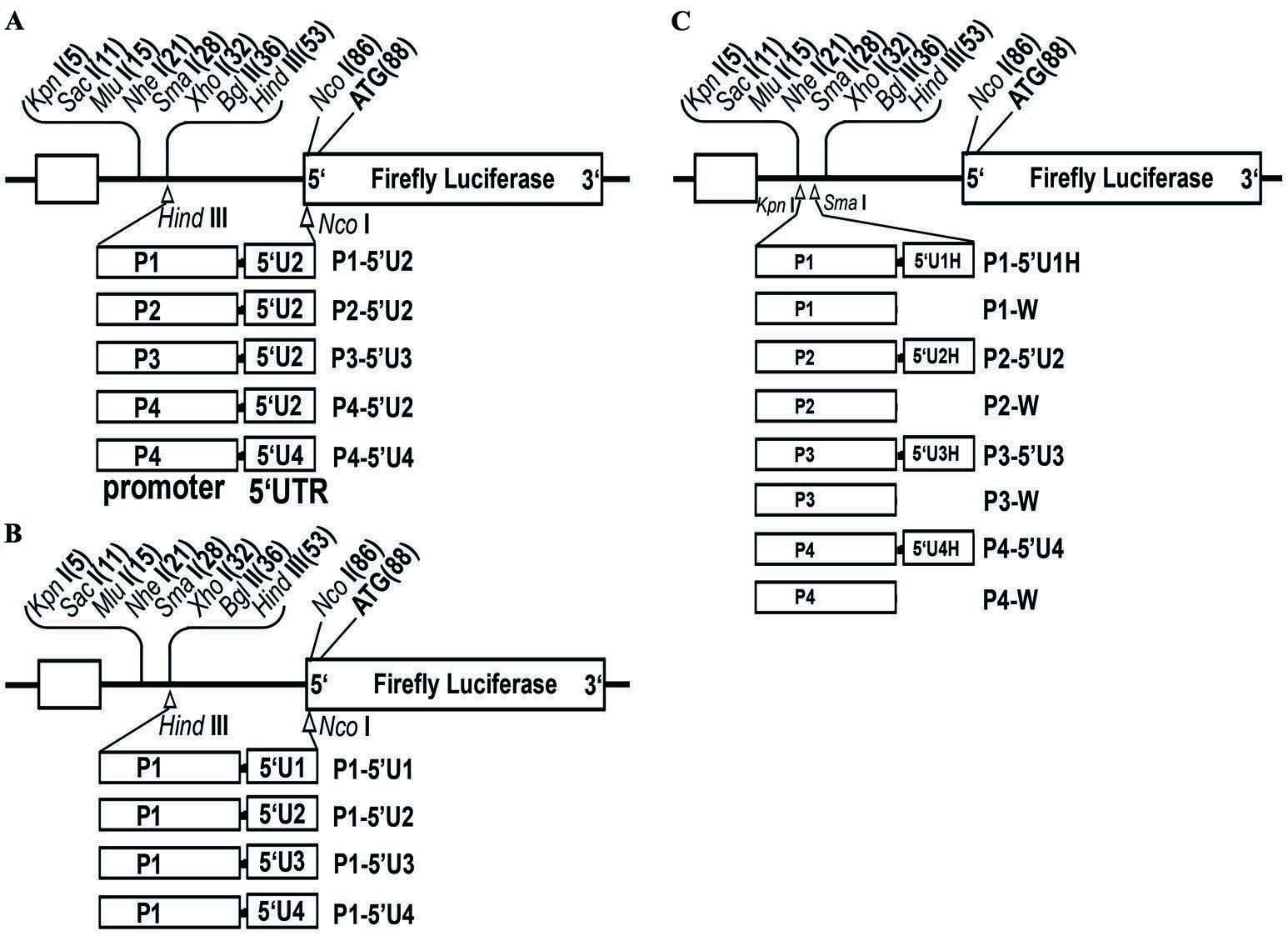

Supplement: Figure S5 — The Sketch Map of Construction of HLA-DRB1 Promoter Vectors. Only the reporter gene and its 5′ flanking region in which the multiple clonal sites are located, were shown on the sketch. The numbers in the parenthesis following the names of restriction endonuclease (REN) denote the relative positions of the RENs at the vector sequence. The rectangles below multiclonal sites represent the fragments of promoters or 5′UTRs from HLA-DRB1 genes, which have been inserted into the REN sites denoted by triangles. Three different kinds of promoter vectors were constructed and shown on A, B and C, respectively. (JPG) [file pone.0025794.s005.jpg]

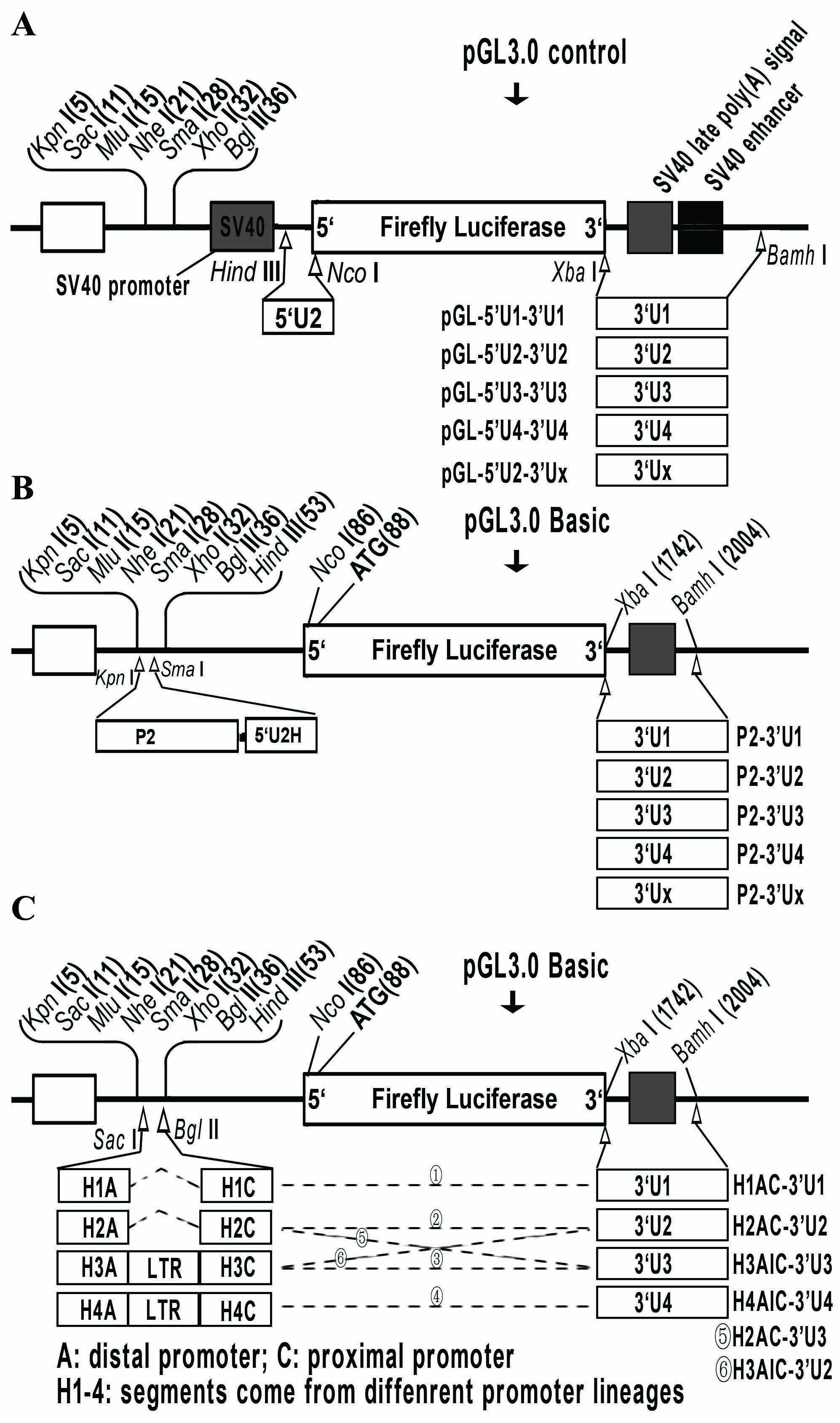

Supplement: Figure S6 — The Sketch Map of Construction of HLA-DRB1 3′UTR and recombinant Vectors. Only the reporter gene and its 5′ flanking region in which the multiple clonal sites are located were shown on the sketch. The numbers in the parenthesis following the names of restriction endonuclease (REN) denote the relative positions of the RENs at the vector sequence. The rectangles below multiclonal sites represent the fragments of promoters or 3′UTRs from HLA-DRB1 genes, which have been inserted into the REN sites denoted by triangles. Three different kinds of vectors were constructed and shown on A, B and C, respectively. (JPG) [file pone.0025794.s006.jpg]
